# Interesting Cases of Intracerebral Haemorrhage Due to Underlying Cerebral Amyloid Angiopathy

**DOI:** 10.7759/cureus.104916

**Published:** 2026-03-09

**Authors:** Ayesha Khan, Richard Marigold

**Affiliations:** 1 Department of Stroke, University Hospital Southampton NHS Foundation Trust, Southampton, GBR

**Keywords:** cerebral amyloid angiopathy, intracerebral haemorrhage, seizures, stroke mimics, transient focal neurological episodes

## Abstract

Cerebral amyloid angiopathy (CAA) is a common small vessel disease occurring in older patients involving amyloid β deposition, which can lead to transient focal neurological symptoms similar to transient ischaemic attacks (TIAs), spontaneous intracerebral lobar haemorrhage and cognitive impairment.

Diagnosis can guide interventions such as blood pressure lowering to reduce future risk of haemorrhage, as well as facilitate decision-making about patients who need to be on antithrombotic medication who are at high thrombotic risk.

Using illustrative cases, we consider the variety of ways in which CAA may present, appropriate investigations and an approach to management.

## Introduction

Cerebral amyloid angiopathy (CAA) is defined by the deposition of amyloid β protein in the walls of small blood vessels in the cerebral cortex and leptomeninges. This can increase the brittleness of the blood vessel wall, increasing the risk of subsequent rupture and resultant haemorrhage. Superficial, peripherally located lobar intracerebral haemorrhage (ICH) is a key clinical feature, often in frail elderly patients with cognitive impairment, with a high mortality and morbidity and recurrence rate of 7.4% [1,2]. This compares with deep intracerebral haemorrhage in the basal ganglia, which is due to underlying arteriosclerosis and cerebral small vessel disease.

Haemorrhage can occur in the subarachnoid space in undulations over the surface of the brain (convexities) (convexity subarachnoid haemorrhage). This can evolve into cortical superficial siderosis, the deposition of haemosiderin within cortical sulci due to subarachnoid haemorrhage from amyloid-affected vasculature, which can be detected on susceptibility weighted (SWI) MRI.

CAA is most commonly found in the occipital lobes, followed by the frontal, temporal and parietal lobes. There is a wide spectrum of clinical presentations, from asymptomatic haemorrhage, progressive cognitive decline and transient focal neurological events to headache, seizures and significant hemiparesis and, in severe cases, major lobar haemorrhage, hydrocephalus, coma and death. Imaging necessitates an MRI with SWI sequences, as a standard CT may appear normal between bleeds and may not detect convexity subarachnoid haemorrhage or cortical siderosis.

## Case presentation

Case 1

A 73-year-old man was admitted to the hospital with three episodes of left face, arm and leg heaviness and numbness with tingling and migratory features lasting 2-3 minutes. He had a background of hypertension and hypercholesterolaemia, and at first, it was thought that these were recurrent transient ischaemic attacks (TIAs). His blood pressure was 130/80 mmHg.

**Figure 1:**
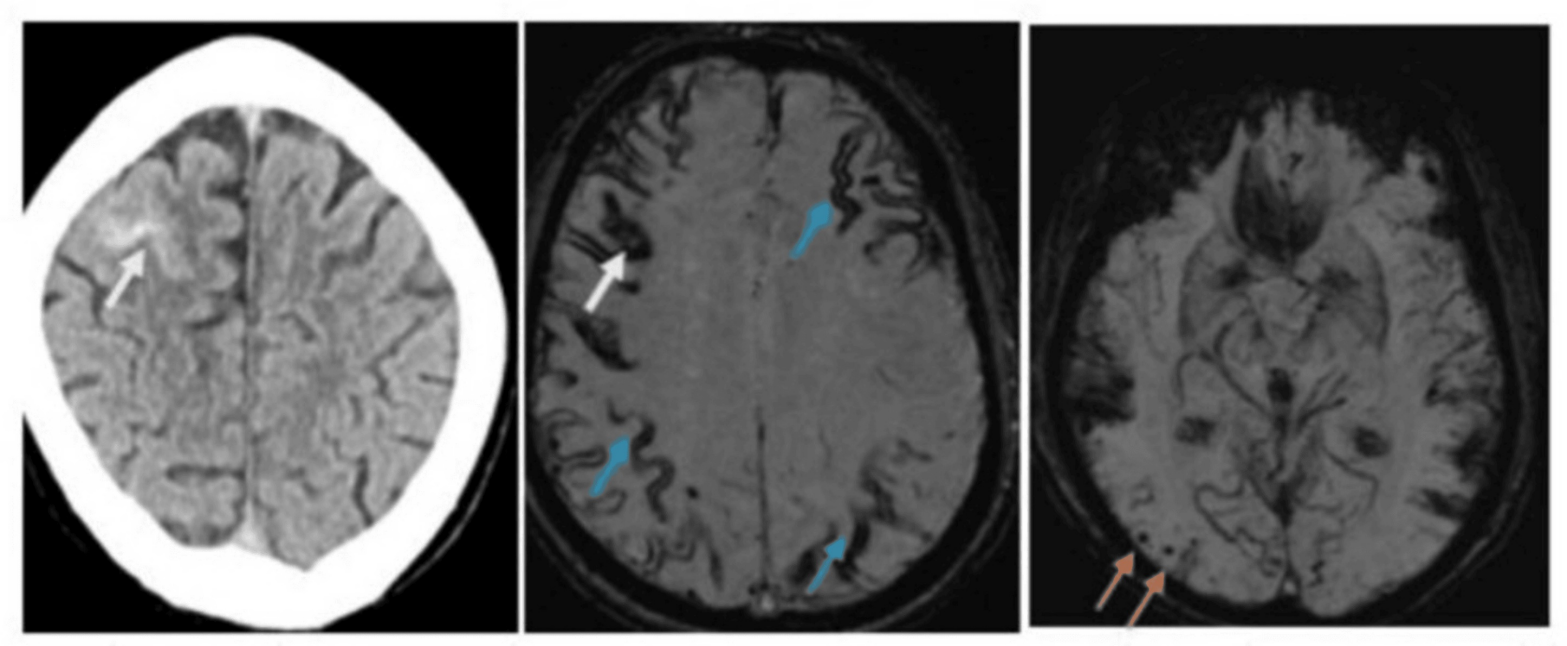
Convexity subarachnoid haemorrhage on non-contrast brain CT confirmed on susceptibility weighted MRI (white arrows) with the presence of cortical siderosis (blue arrows) and multiple lobar microhaemorrhages (orange arrows)

A brain CT scan showed the presence of a right frontal convexity subarachnoid haemorrhage, which was confirmed on MRI with susceptibility weighted imaging with multiple areas of cortical siderosis and lobar microbleeds consistent with CAA (Figure 1).

The differential diagnosis would include transient ischaemic attack (TIA), focal seizure, migraine aura and transient focal neurological episodes (TFNEs). The duration of symptoms is short. TIAs usually last less than an hour, migraine auras 10-30 minutes, focal seizures up to five minutes and transient focal neurological episodes 2-3 minutes. Seizures and migraine tend to start with positive symptoms such as flashing lights, zig-zag lines, arm or leg pain, or paraesthesia or limb jerking movements, whereas TIA and TFNE result in negative symptoms such as loss of limb power, as occurred in this case. Symptoms in TIA migrate over seconds, whereas TFNE spread occurs over minutes [3].

The right frontal convexity subarachnoid haemorrhage (cSAH) and cortical superficial siderosis, with multiple lobar microbleeds on SWI, are characteristic of probable CAA and are strongly associated with TFNE [4].

Case 2

**Figure 2:**
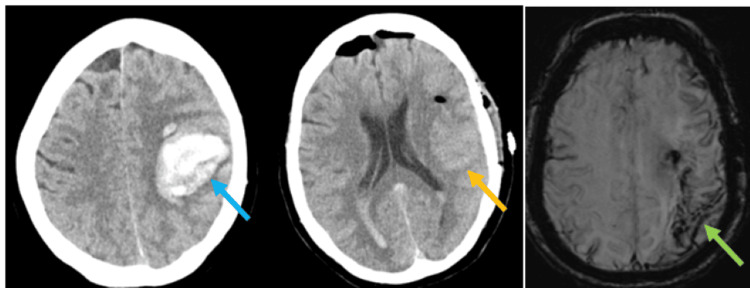
Left temporal lobar haemorrhage (blue arrow) that then underwent haematoma evacuation (orange arrow); the susceptibility weighted MRI shows multiple microbleeds and cortical siderosis in the left parieto-occipital region (green arrow)

A 77-year-old woman who had recently stopped apixaban for a deep vein thrombosis presented to the emergency department in 2019 with a dense right-sided weakness and dysphasia and an NIHSS score of 18, with a pre-existing modified Rankin Scale (mRS) score of 0. Her blood pressure was 195/85 mmHg. Brain CT showed a left temporal haematoma with blood of differing ages in a lobar distribution with no mass effect (Figure 2).

Initial management focused on blood pressure lowering with intravenous labetalol. During the following 24 hours, she became drowsy and comatose, and underwent haematoma evacuation. After a period of rehabilitation, she returned home with aphasia and right-sided weakness, equipment and a package of care, and was discharged with a target blood pressure of 130/80 mmHg.

**Figure 3:**
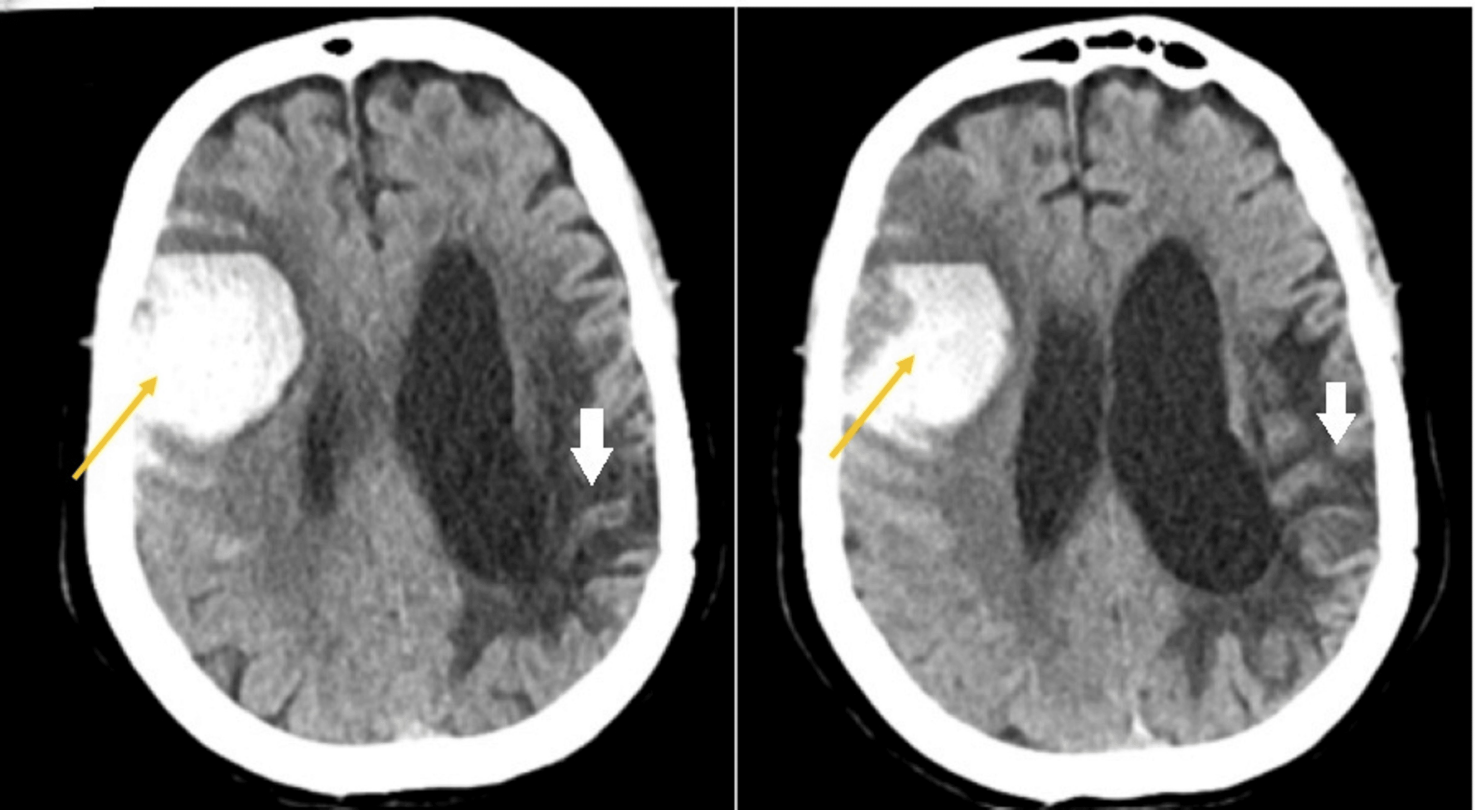
Non-contrast scan showing a further right frontotemporal haemorrhage in 2024; orange arrows show new areas of bleeding, and white arrows show where previous haemorrhage has occurred

Unfortunately, she represented to the hospital in 2024, having collapsed with a dense left-sided weakness. On this occasion, she was found to have a right frontotemporal haemorrhage with surrounding oedema (Figure 3). Palliative care was commenced, and she died later that day.

The pattern of recurrent lobar intracerebral haemorrhage, multiple microbleeds and cortical superficial siderosis fulfils the Boston v2.0 criteria for a diagnosis of sporadic cerebral amyloid angiopathy and higher risk of recurrent haemorrhage.

## Discussion

Clinical spectrum of CAA

Transient Focal Neurological Episodes (TFNEs)

The first case demonstrates transient focal neurological episodes (TFNEs) or amyloid spells due to abnormal electrical activity or cortical spreading depression triggered by recent CAA-related haemorrhage.

In one study, they were the second most common clinical presentation of CAA after intracerebral haemorrhage [5]. Symptoms may be positive with transient paraesthesias of the mouth or hands, which may or may not spread to other body areas, or negative, including focal motor weakness and dysphasia, and some patients experienced migraine-like auras, with flickering, flashing lights and zig-zag lines, or other visual disturbances such as diplopia or transient visual loss. Moreover, 70% had recurrent episodes, which were usually stereotyped (similar to the original presentation) and resolved within 30 minutes [5].

Investigation includes history, examination and blood tests, as well as a brain CT to assess for convexity subarachnoid haemorrhage, and MRI with susceptibility weighted imaging to detect cortical superficial siderosis (cSS) and microbleeds. CT or MR angiography is required to exclude distal vascular malformations or aneurysms [6,7].

Management includes considering blood pressure lowering to <130/80 mmHg to prevent potential haemorrhage progression, pausing antithrombotic drugs where convexity subarachnoid haemorrhage has occurred before restarting, depending on the ongoing antithrombotic indication and limiting to a single antiplatelet [3,6].

Despite a lack of trial evidence, anticonvulsants such as lamotrigine or levetiracetam, or those used in migraine, such as topiramate, often show some benefit, and patients can be reassured that symptoms usually settle in days to weeks and do not represent underlying haemorrhage [5,6].

Acute ICH management in CAA

The mainstay of acute haemorrhage treatment is urgent blood pressure lowering to <140/90 mmHg within an hour of admission to hospital for patients presenting within six hours of onset. Intravenous labetalol, hydralazine and lercandipine are now preferred over glyceryl trinitrate (GTN) as a recent prehospital trial [8] suggested an increase in mortality. If haematoma evacuation occurs as in this case, a tissue sample for histopathological analysis can be helpful in making the diagnosis.

Risk of recurrent haemorrhage

The risk of recurrent haemorrhage is 7.4%-10% per year in cerebral amyloid angiopathy, and increased risk factors include cSAH, cSS, previous lobar ICH and centrum semi-ovale perivascular space severity [1,9]. The risk is also high with TFNE, with one European study suggesting recurrent ICH rates as high as 50% after 14 months [6]. Case 2 highlights the challenges of managing the increased risk of haemorrhage recurrence where anticoagulation is indicated. Alternative treatment options include left atrial appendage occlusion, inferior vena cava (IVC) filter insertion and a shorter duration of anticoagulation [4].

Long-term vascular risk factor management

Tight blood pressure control has been shown to reduce intracerebral haemorrhage recurrence using perindopril [10], and a lower target of 120/80 mmHg has been suggested [4]. Antiplatelet medication through monotherapy, such as aspirin, should only be considered in cases where there is previously known arterial occlusive disease [11].

The International CAA Association and World Stroke Organisation have recently issued a joint scientific statement about managing comorbidities in patients with CAA to reduce the risk of future bleeding [4]. These are outlined in Table 1.

**Table 1:** Recommendations for managing comorbidities in CAA to reduce intracerebral haemorrhage risk CAA: cerebral amyloid angiopathy, IVC: inferior vena cava, NSAIDs: nonsteroidal anti-inflammatory drugs Source: [4,12-16]

Recommendations for managing comorbidities in CAA to reduce intracerebral haemorrhage risk
The following are suggested to manage cardiovascular risk factors
· Hypertension: goal ≤ 130/80 mmHg, some guidelines ≤ 120/80 mmHg
· Hyperglycaemia: maintain euglycaemia
· Hyperlipidaemia: statin use as per guidelines
· Antiplatelets: for established indications only and monotherapy, e.g., ischaemic stroke, myocardial infarction, peripheral vascular disease
· Depression: avoid selective serotonin reuptake inhibitors as they may increase the risk of haemorrhage
· NSAIDS: avoid NSAIDs as the risk of ICH increases
· Alcohol: limit alcohol to less than two drinks per day, as it has been shown to increase blood pressure and bleeding risk
For patients needing anticoagulation
· Non-valvular atrial fibrillation: consider left atrial appendage occlusion as an alternative
· Deep vein thrombosis: consider IVC filter or shorter-term anticoagulation
· Pulmonary embolism: consider shorter-term anticoagulation based on severity
No alternative to anticoagulation
· New left ventricular thrombus: short-interval imaging for early cessation of anticoagulation
· Mechanical valve: consider warfarin if high-risk metal valve (previous valve-related stroke); in aortic mechanical valves with no additional risk factors and lobar ICH, consider antiplatelet therapy
Consider valve replacement with a bioprosthetic valve as appropriate
· Antiphospholipid antibody syndrome: no alternative to long-term anticoagulation
Intravenous thrombolysis
· Significant caution should be taken in deciding whether to treat a patient with intravenous thrombolysis with cerebral amyloid angiopathy
· Endovascular treatment is the preferred option if applicable
· Clinicians can consider intravenous thrombolysis in CAA in the absence of a previous intracerebral haemorrhage, but patients with a prior CAA-related ICH are at increased risk of haemorrhagic complications

## Conclusions

These clinical cases demonstrate the varied and often challenging way in which patients with CAA can present to outpatient clinics in medicine and neurology as suspected seizures or amnesic episodes, and presyncope, or to the emergency department with frank lobar intracerebral haemorrhage.

These cases illustrate how CAA‑related TFNE and recurrent lobar ICH align with current recommendations emphasising strict blood pressure control and cautious use of antithrombotic therapies, but there needs to be a patient-centred individualised management plan, particularly where there is very high thrombotic risk. Broader guideline-based considerations beyond the scope of these cases include a more detailed discussion of thrombolysis, mechanical heart valve and triple-positive antiphospholipid antibody syndrome (APS).
